# Draft Genome Sequences of *Mycoplasma auris* and *Mycoplasma yeatsii*, Two Species of the Ear Canal of *Caprinae*

**DOI:** 10.1128/genomeA.00280-13

**Published:** 2013-06-13

**Authors:** Emilie Dordet-Frisoni, Eric Baranowski, Aurélien Barré, Alain Blanchard, Marc Breton, Carole Couture, Virginie Dupuy, Patrice Gaurivaud, Daniel Jacob, Claire Lemaitre, Lucía Manso-Silván, Macha Nikolski, Laurent-Xavier Nouvel, François Poumarat, Pascal Sirand-Pugnet, Patricia Thébault, Sébastien Theil, François Thiaucourt, Christine Citti, Florence Tardy

**Affiliations:** INRA, UMR 1225, IHAP, Toulouse, Francea; Université de Toulouse, INP, ENVT, UMR 1225, IHAP, Toulouse, Franceb; Université de Bordeaux, Centre de Bioinformatique et Génomique Fonctionnelle, Bordeaux, CBiB, Bordeaux, Francec; INRA, UMR 1332 de Biologie du Fruit et Pathologie, Villenave d’Ornon, Franced; Université de Bordeaux, UMR 1332 de Biologie du Fruit et Pathologie, Villenave d’Ornon, Francee; CIRAD, UMR CMAEE, Montpellier, Francef; INRA, UMR 1309 CMAEE, Montpellier, Franceg; Anses, Laboratoire de Lyon, UMR Mycoplasmoses des Ruminants, Lyon, Franceh; Université de Lyon, VetAgro Sup, UMR Mycoplasmoses des Ruminants, Marcy L’Etoile, Francei; Université Bordeaux, LaBRI, UMR 5800, Talence, Francej

## Abstract

We report here the draft genome sequences of *Mycoplasma auris* and *Mycoplasma yeatsii*, two species commonly isolated from the external ear canal of *Caprinae*.

## GENOME ANNOUNCEMENT

*Mycoplasma auris* and *Mycoplasma yeatsii*, of the hominis and spiroplasma phylogenetic groups, respectively, are two recently described species of the *Mycoplasma* genus (1, 2). Both species appear to have little clinical relevance, yet they are commonly isolated from the external ear canal of *Caprinae* (3–8), often in association with *Mycoplasma agalactiae* (hominis group) and/or *Mycoplasma mycoides* subsp. *capri* (spiroplasma group), two pathogens of small ruminants that are responsible for contagious agalactia (9). Ruminant *Mycoplasma* species of these two remote groups were shown to have exchanged a significant amount of genetic information by horizontal gene transfer (HGT) (10). Thus, the ear canal may offer an ecological niche in which HGT can occur between pathogenic and nonpathogenic *Mycoplasma* species.

Genome sequences are available for *M. agalactiae* and for *M. mycoides* subsp. *capri*, but not for *M. auris* and for *M. yeatsii*. To fill this gap, we report here the complete genome sequence of *M. yeatsii* strain 13926 and *M. auris* strain 15026, isolated in France from ear swabs of a domestic goat (within a flock with a clinical history of mycoplasmosis [7]) and an Alpine ibex (during an abnormal mortality episode with recurrent isolations of *M. agalactiae* [11]), in 2003 and 2008, respectively. Whole-genome sequences were obtained using a combination of Illumina (single reads) and 454 (mate paired with 8-kb insert size), resulting in a 50× (*M. auris*) and 69× (*M. yeatsii*) median coverages. Assembly was performed using Newbler 2.3, and the annotation was conducted using a customized version of the CAAT-Box platform (12) with an automatic preannotation for coding sequences (CDSs) followed by expert validation, as detailed previously (11). Genome analysis and comparisons were mainly conducted using the MolliGen 3.0 platform (13).

Sequence data showed that the estimated genome sizes (767,867 bp and 896,612 bp for *M. auris* and *M. yeatsii*, respectively), the G+C% (27.20 and 25.76% for *M. auris* and *M. yeatsii*, respectively), the gene density (89.13 and 85.24% for *M. auris* and *M. yeatsii*, respectively), and the highest similarity scores of CDSs were consistent with the phylogenetic grouping of these species (2). *M. auris* displayed 26 CDSs (out of 616) with no homolog in ruminant *Mycoplasma* species sequenced so far, one of which has similarities with an *Mycoplasma hominis* CDS annotated as the virulence-associated protein D. Of the 32 *M. yeatsii*-specific CDSs (out of 687), 16 correspond to lipoproteins containing a DUF285 domain, also found in a large family of surface proteins shared by other ruminant *Mycoplasma* species (14). Finally, both genomes displayed sequences related to an integrative conjugative element, with only *M. auris* having a complete element. Altogether, these data indicate that *M. auris* and *M. yeatsii* may be equipped with or able to acquire virulence genes from other *Mycoplasma* species sharing their habitat.

### Nucleotide sequence accession numbers.

These Whole-Genome Shotgun projects have been deposited at DDBJ/EMBL/GenBank under the accession no. AORI00000000 for *M. auris* 15026 and AORK00000000 for *M. yeatsii* 13926. The versions described in this paper are the first versions, accession no. AORI01000000 and AORK01000000, respectively.
